# UV Irradiated Graphene-Based Nanocomposites: Change in the Mechanical Properties by Local HarmoniX Atomic Force Microscopy Detection

**DOI:** 10.3390/ma12060962

**Published:** 2019-03-22

**Authors:** Liberata Guadagno, Carlo Naddeo, Marialuigia Raimondo, Vito Speranza, Roberto Pantani, Annalisa Acquesta, Anna Carangelo, Tullio Monetta

**Affiliations:** 1 Department of Industrial Engineering, University of Salerno, Via Giovanni Paolo II, 132, Salerno, 84084 Fisciano, Italy; cnaddeo@unisa.it (C.N.); mraimondo@unisa.it (M.R.); vsperanza@unisa.it (V.S.); rpantani@unisa.it (R.P.); 2 Department of Chemical Engineering, Materials and Industrial Production, University of Napoli Federico II, Piazzale Tecchio 80, 80125 Napoli, Italy; annalisa.acquesta@unina.it (A.A.); anna.carangelo@unina.it (A.C.); monetta@unina.it (T.M.)

**Keywords:** polymer durability, HarmoniX AFM, epoxy-based coating, photo-oxidation of graphene-based nanocomposites, UV-irradiation, mechanical properties, multiphase polymeric systems

## Abstract

Epoxy based coatings are susceptible to ultra violet (UV) damage and their durability can be significantly reduced in outdoor environments. This paper highlights a relevant property of graphene-based nanoparticles: Graphene Nanoplatelets (GNPs) incorporated in an epoxy-based free-standing film determine a strong decrease of the mechanical damages caused by UV irradiation. The effects of UV light on the morphology and mechanical properties of the solidified nanocharged epoxy films are investigated by Atomic Force Microscopy (AFM), in the acquisition mode “HarmoniX.” Nanometric-resolved maps of the mechanical properties of the multi-phase material evidence that the incorporation of low percentages, between 0.1% and 1.0% by weight, of graphene nanoplatelets (GNPs) in the polymeric film causes a relevant enhancement in the mechanical stability of the irradiated films. The beneficial effect progressively increases with increasing GNP percentage. The paper also highlights the potentiality of AFM microscopy, in the acquisition mode “HarmoniX” for studying multiphase polymeric systems.

## 1. Introduction

Nowadays there is a growing need to prolong the service life of bulk and coating polymeric materials. Solutions in this direction would reduce the supply of resources and make the problem of recycling less burdensome. Carbon nanostructured forms have proven to be able to protect polymeric materials against potential hazards associated with the most common use of polymeric materials, among which UV photooxidation, flammability, mechanical wear and so forth. Unlike traditional nanofillers, carbon nanotubes and graphene-based nanoparticles are able to significantly enhance physical and chemical properties of the resulting nanocomposites also at very low dispersant loading (between 0.01 wt.% and 3 wt.%) [1,2,3,4,5,6,7,8,9,10,11,12,13,14,15,16,17,18,19,20,21,22,23].

The incorporation of Multi wall carbon nanotubes (MWCNTs) in syndiotactic Polypropylene (sPP) films, determines a strong decrease in the rate of photooxidation reactions simultaneously enhancing oxidative thermal stability of the hosting matrix [23,24].

A similar beneficial effect is found for different polymeric materials, such as Polystyrene-Polybutadiene-Polystyrene (SBS) [1], isotactic polypropylene [25], Poly(ethylene-*co*-vinyl acetate) [26], polylactic acid [27], epoxy polymer [28,29,30,31], Thermoplastic Polyurethane (TPU) [32] and so forth. Unlike CNT nanoparticles, the effect of graphene on the UV irradiation of nanocomposites is poorly studied in literature and only some paper is present in the literature on this specific aspect, in spite of the relevant interest from a practical point of view. In fact, graphene nanoparticles have proven to be ideal candidates for improving many properties of epoxy nanocomposites both as coating and bulk materials, among these: (a) improvement in the electrical conductivity [3]; (b) adhesion properties [33]; (c) protection against lightning strike and icing and so forth [34,35,36]. The possibility to combine these functions with increased durability of the materials is strongly desired in several practical applications. Preliminary studies are already available in the literature. Epoxy-Graphene (E/G) nanocomposites with different loading of graphene were prepared via in situ pre-polymerization by Alhumade et al. [37] The prepolymer/graphene mixture was spin coated on SS304 substrate and after thermal curing was analysed to investigate the effect of graphene on different properties of the coating. The incorporation of graphene was found able to enhance mechanical properties, thermal properties as well as the adhesion of the epoxy composite to the SS304 substrate. The coated filled and unfilled samples were exposed to UV irradiation and the first morphological observations confirmed enhanced resistance to UV irradiation. Ghasemi-Kahrizsangi et al. found an improvement in the UV degradation resistance of epoxy coatings using modified carbon black nanoparticles [38]. Nanostructured forms of carbon seem to have the great potential to enhance UV resistance of the hosting polymeric matrices.

Furthermore, considering the similarity between CNT walls and graphene sheets, the beneficial effects on the durability of nanocomposites due to embedded graphene nanoparticles are expected. In several applications, graphene-based nanocomposites are potentially designed for outdoor applications or as the outer layer of complex parts making them exposed to ultra violet irradiation, hence prone to UV degradation. The more relevant damages, from an applicative point of view, are most of all related to the degradation in the mechanical properties caused by the UV irradiation. Widely diffused polymers (PE, iPP, sPP etc.) in the absence of UV stabilizers or specific nanofillers, show mechanical degradations of non-trivial entity in relatively brief periods. For this reason, the possibility to use a technique, which allows monitoring the mechanical properties of the sensitive part (more subject to UV degradation) of a multiphase polymeric material is strongly desired. This manuscript focuses on showing the possibility of extending traditional Atomic Force Microscopy (AFM) imaging with a technique, which is sensitive to the punctual changing in the mechanical properties of the surface film, providing information on the heterogeneity of polyphasic materials. The polyharmonic response varies with changing local mechanical properties. The comparison of the material properties on identified regions, before and after UV irradiation, provides relevant information on the local changes caused by the photooxidative degradation. In particular, AFM microscopy, in the acquisition method “HarmoniX,” has been employed to study both qualitative and quantitative nanometric-resolved maps of the mechanical properties. It is worth noting that, this non usual AFM acquisition method has been employed because, in the conventional TM-AFM, commonly used for probing the topography and composition of surfaces by adopting the acquired phase maps [39], it is difficult to relate the phase differences to the actual quantitative mechanical properties due to the fact that they depend on a mixture of elastic, adhesive and dissipative properties of the sample and of the AFM imaging parameters, such as set point, drive amplitude or the ratio of the free air amplitude to the drive amplitude [40]. Therefore, by coupling the AFM phase maps with the AFM modulus maps, morphologies and distribution of crystalline aggregates (for both samples, with and without the addition of GNPs) can be easily recognized. Furthermore, the effect of UV irradiation on the local mechanical damages of the film can be easy detected. Samples not containing GNPs highlight a strong mechanical degradation, in terms of elastic modulus, of the polymeric matrix after the UV irradiation. The incorporation of graphene nanoplatelets (GNPs) in the polymeric film causes a strong increase in the mechanical stability of the irradiated films. The entity of this beneficial effect on the mechanical properties progressively increases with increasing GNP percentage.

## 2. Materials and Methods

### 2.1. Materials

Waterborne epoxy resin (Wapex 660, Sikkens, Akzo Nobel Coatings SpA, Novara, Italy) from AkzoNobel is composed of 63 wt.% of the solid content (as per TiO_2_, BaSO_4_, CaCO_3_). It is a bi-component commercial waterborne resin (A and B components), without corrosion inhibitors.

Graphene nanoplates (GNPs) were purchased by Cometox (Milan, Italy). The surface area of the GNPs is 500 m^2^/g (according to the manufacturer).

#### Preparation of the Unfilled and Filled Epoxy Based films

Epoxy films were obtained by embedding 0.1, 0.5 and 1 wt.% of GNPs in the component A of the Wapex 660 resin. GNPs were dispersed in the resin A at room temperature using an ultrasonic bath with a frequency of 50 Hz for 20 min. Then, the hardener (component B of epoxy resin) was added to the blend and mixed for further 20 min by using a mechanical stirrer. The films have been realized by spreading the epoxy resin, with and without the addition of GNPs, on a teflon plate by means of a doctor blade. Then, they were cured in an oven at 150 °C for 10 min to obtain a thickness of about 30 ± 1.5 µm and stored in drying box after complete curing. Unfilled samples were prepared by mixing the component A and B of the resin at room temperature [41]. The samples were 100 × 100 mm^2^ in dimension.

In this paper, for simplifying the name of the samples, the sample without GNPs and then with only the solid content (as per TiO_2_, BaSO_4_, CaCO_3_) is named unfilled sample (0GNPs sample), whereas the samples with 0.1, 0.5 and 1 wt.% of GNPs are named with the acronyms 0.1GNPs, 0.5GNPs, 1GNPs. The sample subjected to 550 h of UV irradiance are named 0GNPs (550), 0.1GNPs (550), 0.5GNPs (550), 1GNPs (550). The acronyms of the developed samples, together with information on their composition, are listed in Table 1.

### 2.2. Methods

Energy dispersive X-ray (EDX) analysis was performed using an Energy Dispersive X-ray analyser (EDX model: INCA Energy 350, Oxford Instruments, Witney, UK), using the signal of sodium atoms. Before the evaluation of the elemental composition, the samples were coated with chromium (layer thickness 150 Ǻ) using a turbo sputter coater (model: K575X, EmiTech Ashford, Kent, UK).

Morphological analysis of the GNP sample was obtained using Scanning Electron Microscope-SEM (model: LEO 1525, Carl Zeiss SMTAG, Oberkochen, Germany). The sample was placed on a carbon tab previously stuck to an aluminium stub and covered with a 250 Å-thick gold film using a sputter coater Agar model: 108 A, (Agar Scientific, Stansted, UK), before being subjected to morphological analysis.

X-ray Diffraction (XRD) analysis was carried out using a Bruker D8 Advance diffractometer (Bruker Axs Inc., Madison, WI, USA) with Ni-filtered CuKα radiation (λ = 1.54050 Å).

#### HarmoniX AFM Characterization

AFM analyses were performed by a NanoScope MultiMode V scanning probe microscope (Veeco, Santa Barbara, CA, USA) equipped with HarmoniX tool. Tests were performed with HMX probe silicon cantilevers (Bruker, Billerica, MA, USA) with nominal radii of c.a. 10 nm. The cantilever oscillation is composed of two different movements, torsional and vertical. These movements have different frequencies, in particular the amplitude frequency of the torsional movement is higher than the tapping frequency [42,43].

The reconstruction of sample morphology is due to the vertical movements in standard tapping mode, whereas, the reconstruction of elastic modulus maps is due to the tip sample force interactions during the torsional movement [42]. Lastly, the AFM elastic modulus values were obtained averaging the elastic moduli on the investigated area by the Nanoscope software version 7.30.

HarmoniX measurements were done in air. Cantilevers were calibrated using a standard polystyrene/low density polyethylene (PS/LDPE) sample. The adopted vertical frequency was 49 kHz and the torsional frequency was 1044 kHz. Imaging was performed with 0.5 Hz scan rates, considering 20 harmonics.

AFM acquisitions were performed by adopting the HMX-10 probe with a nominal tip radius of 10 nm. This probe allows mapping material mechanical property of samples in the 10 MPa to 10 GPa range. Positions on the sample surface were evaluated with an accuracy of the 10 nm, whereas the accuracy of the elastic modulus is 10 MPa.

Image processing and data analysis were performed with the NanoScope software version 7.30 and NanoScope Analysis version 1.80. The NanoScope software gives elastic modulus maps by elaborating HarmoniX AFM imaging through Derjaguin-Muller-Toporov (DMT) model [44].

The photo-oxidative degradation was carried out by exposing filled and unfilled samples (30 ± 1.5 thick film) to UV-A radiation, reproducing the ultraviolet (295–380 nm) component of solar radiation at the earth surface. This treatment was carried out by an Accelerated Weathering Tester model. QUV/spray (Q-Panel Lab Products, Cleveland, OH, USA) with Solar Eye Irradiance Control System and fluorescent UV-A lamps (UVA-340).

## 3. Results and Discussion

### 3.1. Structural and Morphological Investigation

#### 3.1.1. X-ray Investigation

**Figure 1:**
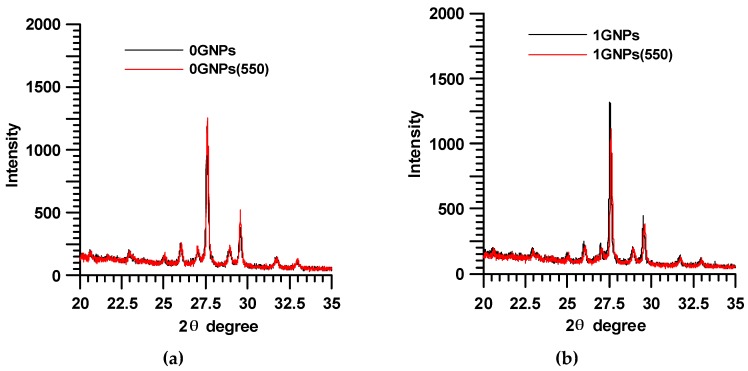
X-ray diffraction patterns of the samples 0GNPs (black trace) and 0GNPs (550) (red trace) (**a**); and 1GNPs and 1GNPs (550) (**b**).

Figure 1 shows the X-ray diffraction patterns of the sample without GNPs, before (0GNPs-black trace) and after UV treatment (0GNPs (550)—red trace) and the sample with 1 wt.% of GNPs, before (1GNPs-black trace) and after UV treatment (1GNPs (550)—red trace). X-ray spectra show the sharp reflections of TiO_2_, BaSO_4_, CaCO_3_ crystals, which are superimposed to the amorphous halo of the epoxy matrix. In the spectra of 1GNPs and 1GNPs (550), the reflection 002 of the GNPs at 2θ = 26.2° is not clearly visible due to the small number of GNPs and the intense signals of TiO_2_ and BaSO_4_.

The comparison of the RX spectra before and after the UV treatment clearly highlights that no changes in the crystallographic modification occur after the UV treatment. Furthermore, also the dimensions of the crystallites seem to be almost the same.

#### 3.1.2. Energy Dispersive X-ray (EDX) Analysis

**Figure 2:**
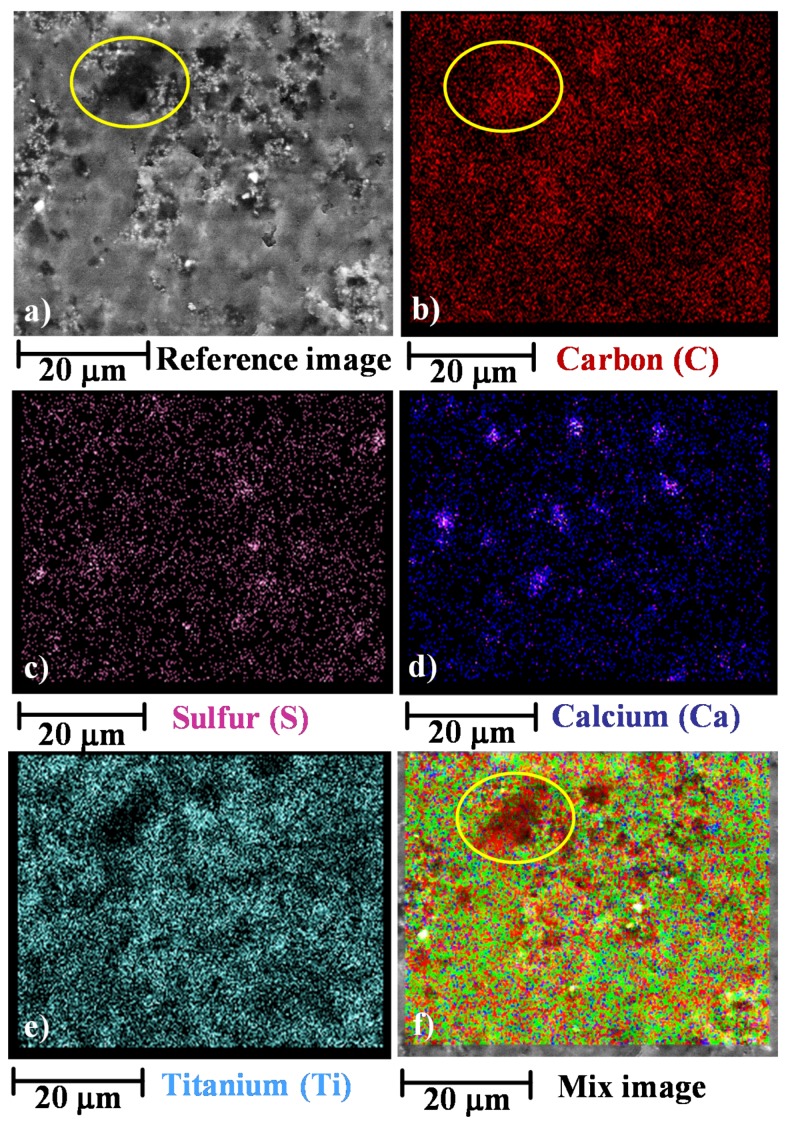
Energy dispersive X-ray (EDX) performed on the fracture surface of the sample 01GNPs: (**a**) reference image; (**b**) map distribution of C; (**c**) map distribution of S; (**d**) map distribution of Ca; (**e**) map distribution of Ti; (**f**) mix image.

The chemical composition of the film surfaces, at level of microscopic spatial domains, has been investigated using the Energy dispersive X-ray (EDX) analysis. The EDX images in Figure 2 show the distribution of C, S, Ca and Ti on the surface of the sample 01GNPs. In particular, the element S (see Figure 2c) is present in the form of BaSO_4,_ whereas the elements Ca (see Figure 2d) and Ti (see Figure 2e) are present in the form of CaCO_3_ and TiO_2_ respectively, as described in the Section 2.1.

From these images, it is quite evident that the crystalline domains corresponding to BaSO_4_, CaCO_3_ and TiO_2_ crystals are well distributed in all the sample. It is worth noting that, although the Carbon element belongs not only to GNPs but also to the hosting matrix (in this last case combined with hydrogen and oxygen), it is possible identify regions where its concentration is higher than the surrounding regions (as an instance see the regions delimitated by the yellow ellipses in Figure 2a, which is the reference image and Figure 2b which shows the Carbon element distribution). This is a clear evidence that these regions are related to the GNPs presence. The mix image shown in Figure 2f, highlights this observation even in a more evident manner (see yellow ellipse).

Starting from this observation, it is rather obvious that the Mix image of Figure 2f (corresponding to the map of all the detected elements) provides a very clear map of the distribution of GNPs on the surface of the film.

**Figure 3:**
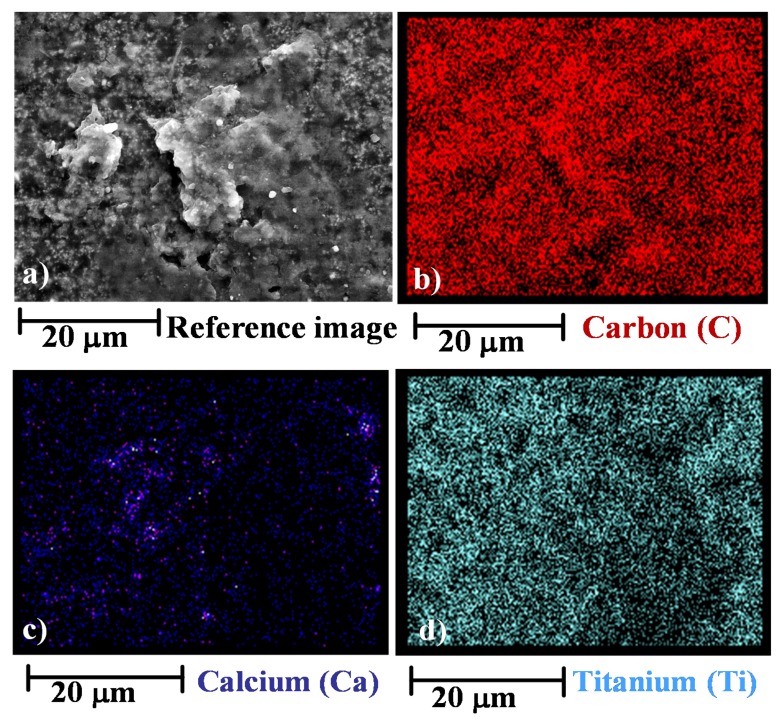
Energy dispersive X-ray (EDX) performed on the fracture surface of the sample 01GNPs (550): (**a**) reference image; (**b**) map distribution of C; (**c**) map distribution of Ca; (**d**) map distribution of Ti.

The EDX images in Figure 3 show the distribution of C, Ca and Ti on the surface of the sample 01GNPs (550).

The EDX images of the irradiated sample seems to highlight a more intense red colour in different regions (see Figure 3b) with respect to Figure 2b. This is an expected result and it is most likely due to the consumption of the resin surrounding the GNPs. In fact, as already evidenced by SEM investigation, the UV treatment can determine, from a morphological point of view, effects very similar to those caused by chemical etching procedures [45]. Furthermore, the distribution of the crystals (BaSO_4_ and CaCO_3_) seems to highlight that the UV radiation determines a disaggregation of the salt crystals as a direct consequence of a little consumption of the hosting matrix between the crystallites. As an instance, if a comparison between Figure 3c, corresponding to the map image of Ca element in the sample 01GNPs (550) and Figure 2d corresponding to the map image of Ca element in the sample 01GNPs is performed, it is clearly that in the case of irradiated sample a partial crystal disaggregation is observed. Contrary to the BaSO_4_ and CaCO_3_ crystallites, the crystalline phase corresponding to TiO_2_ seems to be distributed in the same way both in the untreated and UV irradiated sample.

**Figure 4:**
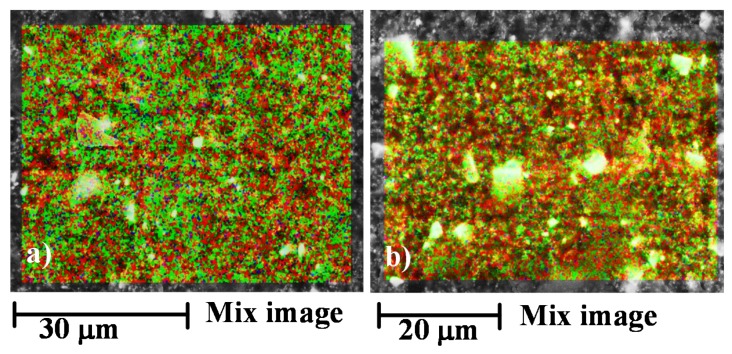
Energy dispersive X-ray (EDX) performed on the fracture surface of the sample 1GNPs before and after UV irradiation: (**a**) mix image of 1GNPs sample; (**b**) mix image of 1GNPs (550) sample.

Very similar results are obtained with the samples 1GNPs and 1GNPs (550), also in this case the irradiated sample seems to highlight a more intense red colour in different regions (see Figure 4a,b) due to the consumption of the thick layer of resin surrounding the GNPs.

#### 3.1.3. SEM Investigation

**Figure 5:**
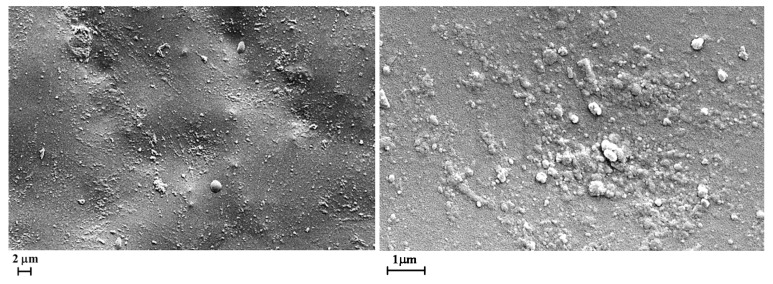
Scanning electron microscopy (SEM) micrographs of the unfilled film before ultra-violet (UV) irradiance (0GNPs sample).

**Figure 6:**
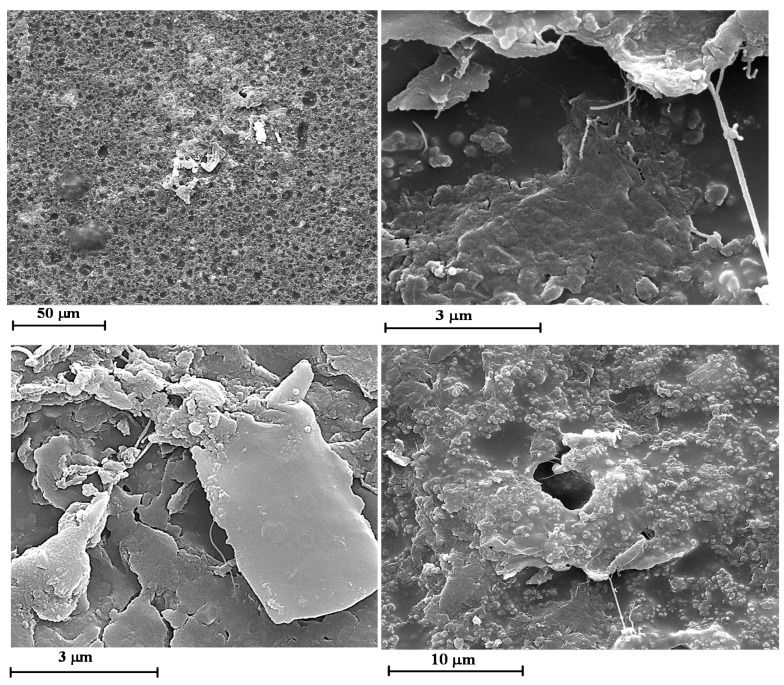
SEM micrographs of the unfilled films after 550 h of UV irradiance (0GNPs (550) sample) at different magnifications.

In order to investigate the influence of the UV irradiation on the morphological features of the films, SEM images of the samples without and with GNPs have been compared. Figure 5 and Figure 6 show the SEM micrographs of the unfilled film before (Figure 5) and after 550 h of UV irradiance (Figure 6) at different magnifications.

The surface of the irradiated sample appears strongly pitted (see image on the top). The micrographs at higher magnification evidence that many parts of the polymer matrix are severely damaged showing deep holes and furrows in the sample. Furthermore, many geometrical o spherical domains due to the crystalline phase of TiO_2_, BaSO_4_, CaCO_3_ are foreseen.

**Figure 7:**
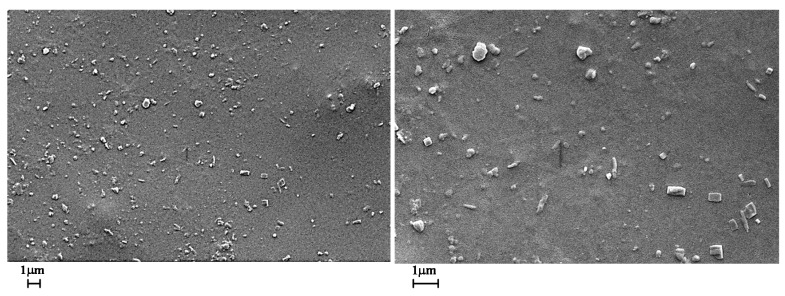
SEM micrographs of the film loaded with 0.1 wt.% of GNPs before UV irradiance (01GNPs sample) at different magnifications.

**Figure 8:**
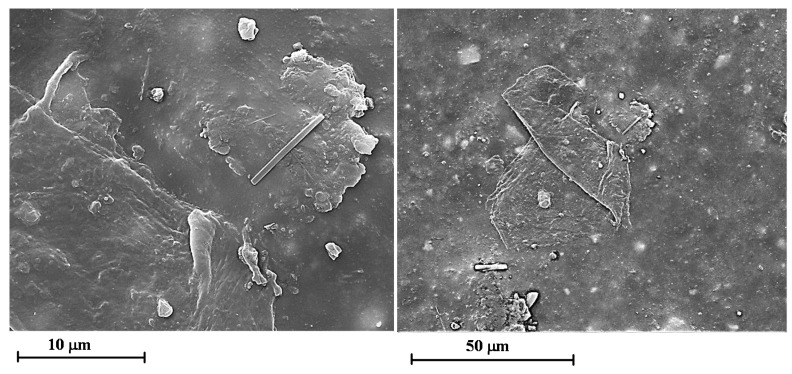
SEM micrographs of the film loaded with 0.1 wt.% of GNPs after 550 h of UV irradiance (01GNPs (550) sample) at different magnifications.

It is worth noting that the most damaged areas belong to the polymer matrix; in fact, as it can be observed in Figure 1, the X-ray profile of the peaks is almost the same before and after the UV treatment. This is a clear indication that the damages do not occur in the crystalline phase but, as expected, in the epoxy matrix, which is an amorphous phase. Figure 7 and Figure 8 show the SEM micrographs of the film loaded with 0.1 wt.% of GNPs before (Figure 7) and after 550 h of UV irradiance (Figure 8) at different magnifications.

The SEM images clearly evidence that a small amount of GNP nanoparticles (0.1 wt.%) prevents or strongly decreases the damages of the sample surface due to the UV irradiation. In fact, the surface of the sample irradiated 550 h does not appear pitted and deeply furrowed like the sample 0GNPs (550) (without GNPs). In the sample after the UV irradiation, GNP nanoparticles are easily observable in the polymer matrix because the UV treatment partially consumes the thin polymer covering the nanoparticles.

**Figure 9:**
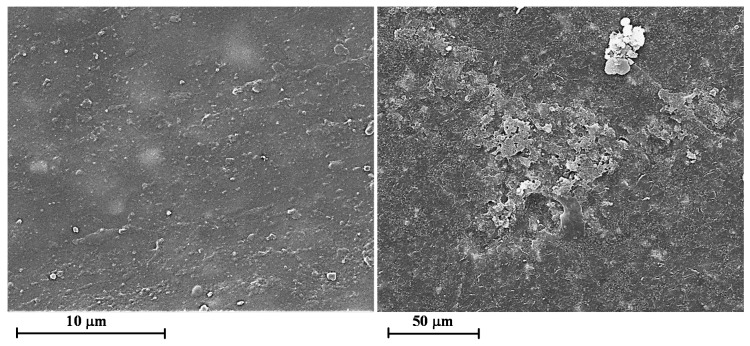
SEM micrographs of the films 1GNPs (on the left side) and 1GNPs (550) (on the right side) at different magnifications.

Figure 9 shows the SEM micrographs of the film loaded with 1.0 wt.% of GNPs before (on the left side) and after 550 h of UV irradiance (on the right side) at different magnifications. Also in this case, the sample containing GNPs seems to be less damaged by the UV irradiation.

Similar results have been observed for all the analysed percentages of GNPs.

The strong stabilizing effect of the GNPs against the UV damage can be due to several factors, which require extended series of experiments to deeply understand their influence on the chemical and physical mechanisms responsible of the UV damage reduction/attenuation. In particular, it is well known that, due to the UV irradiation, in the first stage of the sample photodegradation (both in the thermoplastic and thermosetting matrices), free radicals are formed [46,47,48,49]. After the first stage of radical formation, photooxidation reactions occur. In particular, the oxidation reactions are affected by the availability of the oxygen in the matrix, which is expected to influence the kinetic of the photooxidation reactions and their extend through the matrix. Concerning the availability of oxygen in the matrix and in general the transport properties of gases in polymeric matrices incorporating carbon-based nanofillers, it has been found that GNPs and GO-derived nanoparticles, can significantly reduce gas permeation through the polymer nanocomposite with respect to the neat matrix [50]. A network of nanoplatelets through the polymeric matrix can provide ‘tortuous paths,’ which inhibit or slow down the molecular diffusion in the hosting matrix, thus resulting in significantly reduced permeability [51]. Studies of permeability on chemically modified graphene (CMG) platelets in polystyrene (CMG/PS) nanocomposites suggest that at low loadings (below 0.05 vol.%), the decrease in permeability of the nanocomposite is due to a reduction in gas solubility in the composite; furthermore, the effects of diffusion become even more relevant at higher amount of nanofillers [52]. Studies of permeability on GNPs and GO-derived nanofillers in several polymers have been performed by different authors [50,52,53,54,55,56,57] and it has been found that the nanoparticles strongly affect the transport properties of the resulting nanocomposites; as an instance, in the case of a thermoplastic matrix, polypropylene (PP) loaded with 6.5 of GNP wt.%, the results highlight a 20% reduction in oxygen permeability [58].

Considering that the oxygen plays a very relevant role in the photooxidation of the polymeric matrix, it is very likely that the stabilizing effect against the UV irradiation and therefore the strong decrease in the entity of the damages of the samples is due to a percolating network of ‘tortuous paths’ which inhibits molecular diffusion through the matrix, thus resulting in significantly reduced permeability of oxygen through the matrix. It is most likely that the reduced amount of oxygen is responsible for the strong decrease of the UV damages. Studies concerning the transport properties (of gas and vapours) in the formulated nanocomposites and the kinetic of the photooxidation reactions through FT/IR experiments are still ongoing. They will be described and discussed in a forthcoming paper.

#### 3.1.4. Morphological Investigation through HarmoniX Measurements

**Figure 10:**
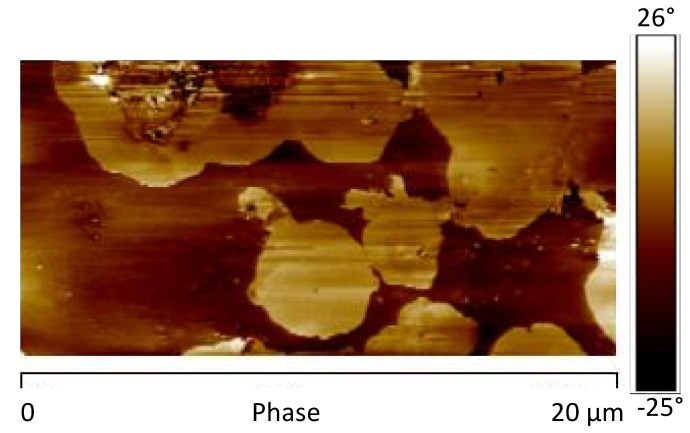
Atomic force microscopy (AFM) phase map image of the sample 0GNPs.

The AFM phase map image of the sample (without GNPs) before the UV irradiation is shown in Figure 10.

AFM phase map image highlights, on the sample surface, the presence of brighter areas, which correspond to the presence of domains characterized by different mechanical response. In particular, isolated circular structures, with diameter of about 3–4 μm, can be detected. They are most likely due to crystalline domains composed of TiO_2_, BaSO_4_ and CaCO_3_.

**Figure 11:**
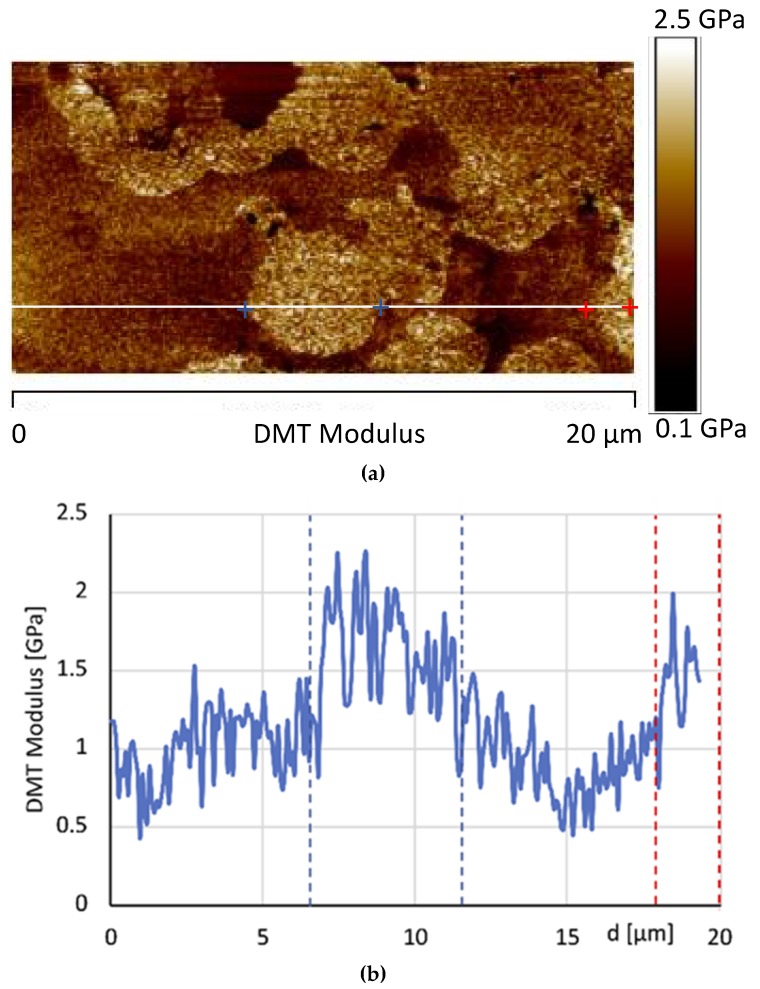
AFM image of the Derjaguin-Muller-Toporov (DMT) modulus maps of the sample 0GNPs (**a**) and AFM image of the DMT modulus maps of the sample 0GNPs with the profile of the value of the elastic modulus (elastic modulus scale of the sample taken along the white line) (**b**).

**Figure 12:**
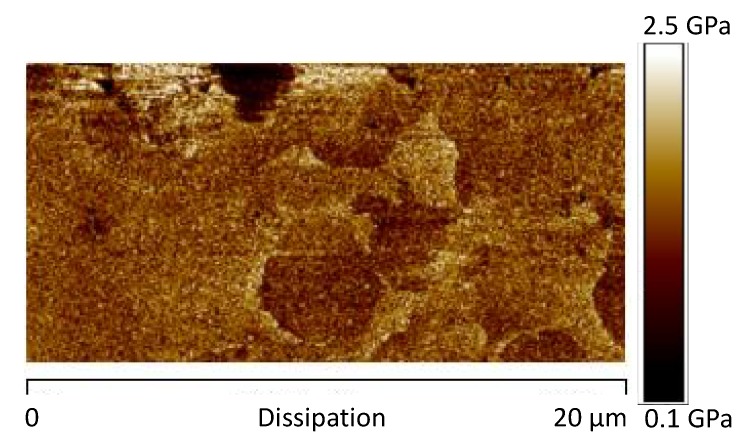
AFM dissipation image of the sample 0GNPs.

Figure 11a, shows the image of the DMT modulus maps of the same sample. As expected, in correspondence of the areas characterized by high phase values, higher levels of the DMT elastic modulus are detected. Moreover, the dissipation image, shown in Figure 12, highlights lower values in correspondence of the brighter areas of the phase map. These observations are consistent with the attribution of the brighter regions to crystalline domains. Figure 11b shows the AFM image of the DMT modulus maps of the sample 0GNPs with the profile of the value of the elastic modulus (elastic modulus scale of the sample taken along the white line). The profile clearly highlights that the value of elastic modulus on the spherical structures is approximately 1.5–2.0 times that detected on the matrix. Furthermore, the brighter regions corresponding to the crystalline domains appear inhomogeneous in the colour, being punctuated by darker areas. This is a clearly evidence that these regions correspond to crystalline aggregates. This last observation is consistent with the noise detected in the profile of the value of the elastic modulus along the white line corresponding to the brighter regions of the crystalline phase (see Figure 11b).

**Figure 13:**
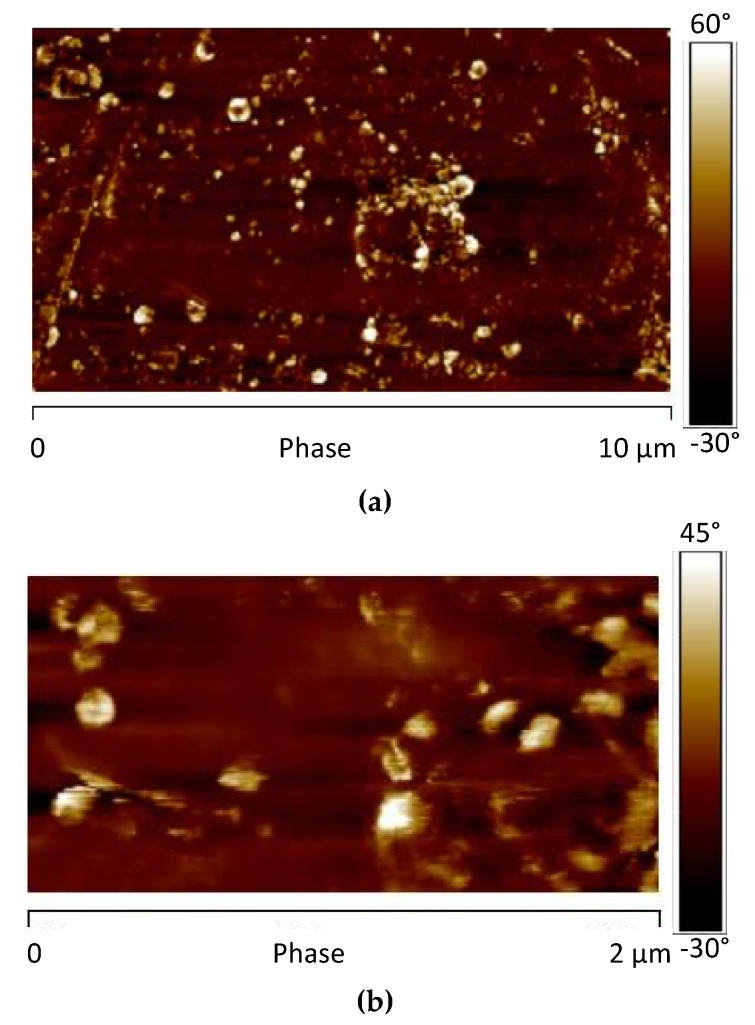
AFM phase map image of the sample 0GNPs (550) acquired adopting a scan size of 10 μm (**a**) and 2.0 μm (**b**).

**Figure 14:**
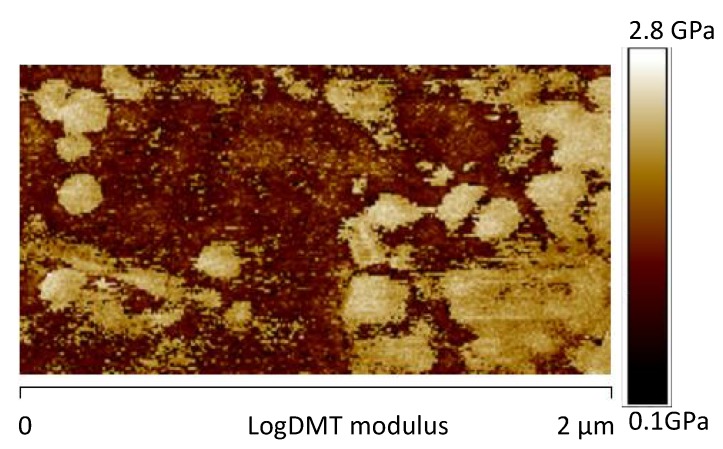
AFM image of the logDMT modulus maps of the sample 0GNPs (550).

**Figure 15:**
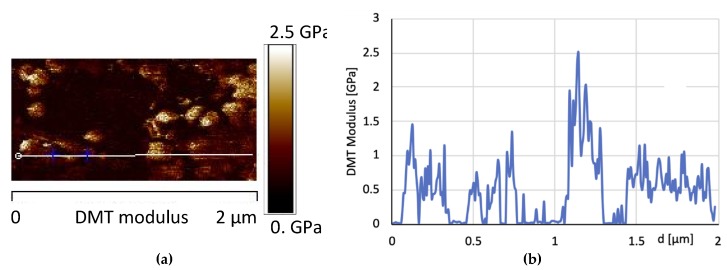
(**a**) AFM image of the DMT modulus maps of the sample 0GNPs (550) with the profile (**b**) of the value of the elastic modulus (elastic modulus scale of the sample taken along the white line).

The AFM phase map images of the sample 0GNPs (550) acquired adopting the scan sizes of 10 μm (Figure 13a) and 2.0 μm (Figure 13b) are shown in Figure 13. In these images, the spherical structures of 3–4 μm, observed in Figure 10 for the unirradiated sample, disappear. They are replaced by isolated brighter structures and aggregates with reduced diameters. These structures and their dimensions are more clearly observed in the AFM image of the logDMT modulus maps of Figure 14, where the dimensions of these isolated structures range between 100 nm and 200 nm. Figure 15 shows the AFM image of the DMT modulus maps of the sample 0GNPs (550) (Figure 15a) with the profile of the value of the elastic modulus (elastic modulus scale of the sample taken along the white line) (Figure 15b). The profile confirms the dimensions of the crystalline domains. Considering the results of the RX investigation, which clearly indicates that no changes in the crystallographic modifications occur and the dimensions of the crystals are almost the same before and after the UV irradiation, it is clear that the UV radiation causes a disaggregation of crystalline aggregates. This result is expected in light of the observations made on the SEM images of Figure 6, where it is evident the consumption of the matrix in many regions around the small crystallites. In particular, the UV radiation, around the crystalline domains, acts as an etching treatment, making the crystallites much more visible.

Concerning the local mechanical properties, the values of the DMT modulus (see the elastic modulus scale of the sample taken along the white line in Figure 15) of the sample 0GNPs (550) were found lower on the polymeric matrix with respect to those detected on the matrix of the unirradiated sample 0GNPs. This result is due to the damages caused on the matrix by the UV irradiation, which acts through two main effects on the polymeric matrix of the sample. Firstly, it consumes the polymer around the crystallites making them more visible and distanced. Secondly, the elastic modulus of the sample decreases; in particular, the modulus of the polymeric matrix hosting the crystallites is significantly reduced with respect to that of the initial sample.

**Figure 16:**
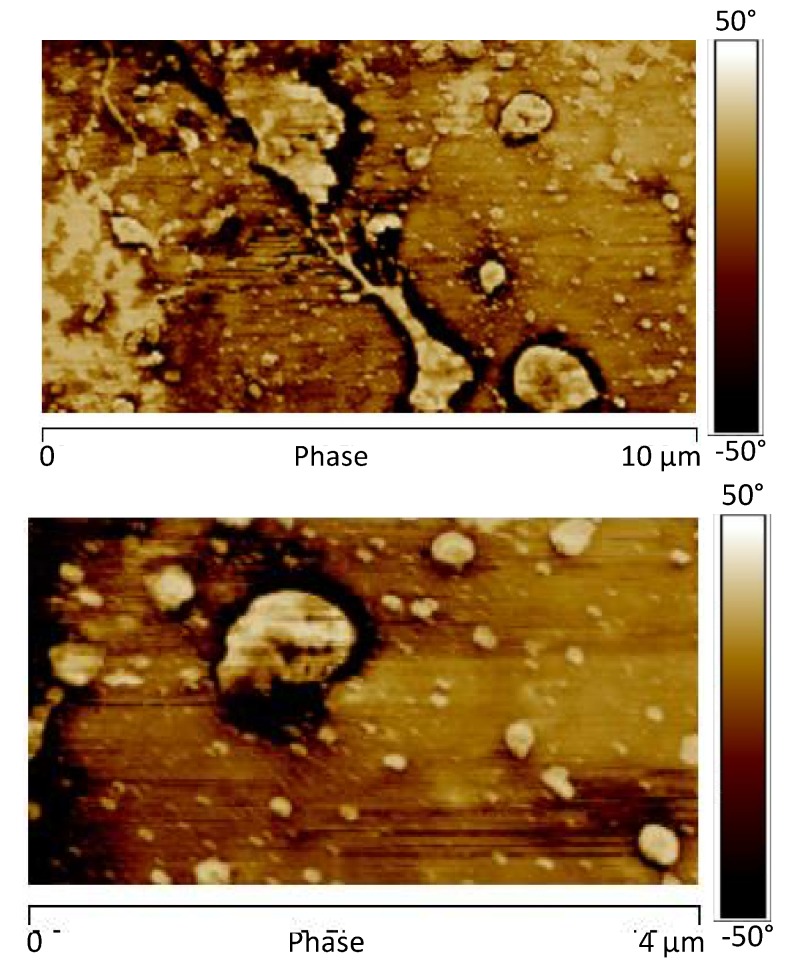
AFM phase map images at two different scan size of the sample 1GNPs.

**Figure 17:**
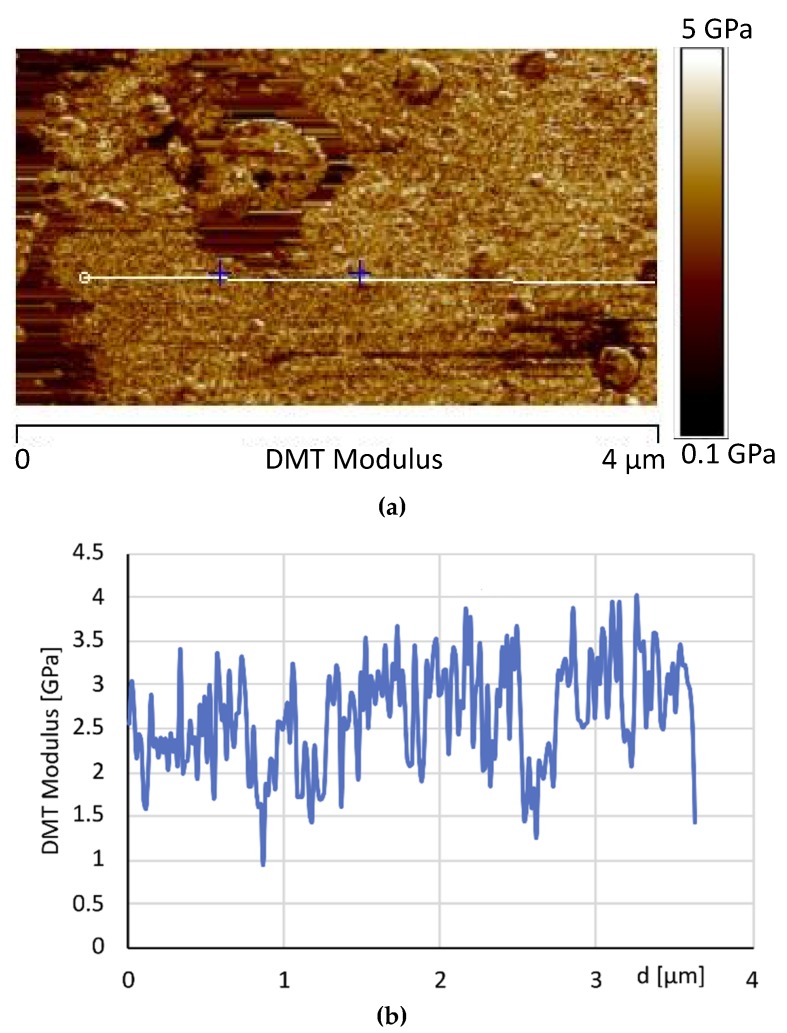
(**a**) AFM image of the DMT modulus maps with the profile (**b**) of the value of the elastic modulus (elastic modulus scale of the sample taken along the white line) of the same sample.

Figure 16 shows the AFM phase map images at two different scan size of the sample 1GNPs, whereas Figure 17 shows the AFM image of the DMT modulus maps with the profile of the value of the elastic modulus (elastic modulus scale of the sample taken along the white line) of the same sample. In Figure 16, small crystalline domains of about 200 nm, together GNPs can be detected; most likely, due to the ultrasonic process performed to disperse GNPs in the polymeric matrix, also the crystallites of TiO_2_, BaSO_4_ and CaCO_3_ are better dispersed.

Both the map image and the profile of the elastic modulus confirm that by adding 1% of graphene, an enhancement of the modulus is observed with respect to the sample 0GNPs. Furthermore, the profile of the value of the elastic modulus (elastic modulus scale of the sample taken along the white line) shows that the modulus distribution on 1GNP sample is more uniform with respect to that detected for the sample 0GNP. This result is due to the presence of GNPs in the film.

**Figure 18:**
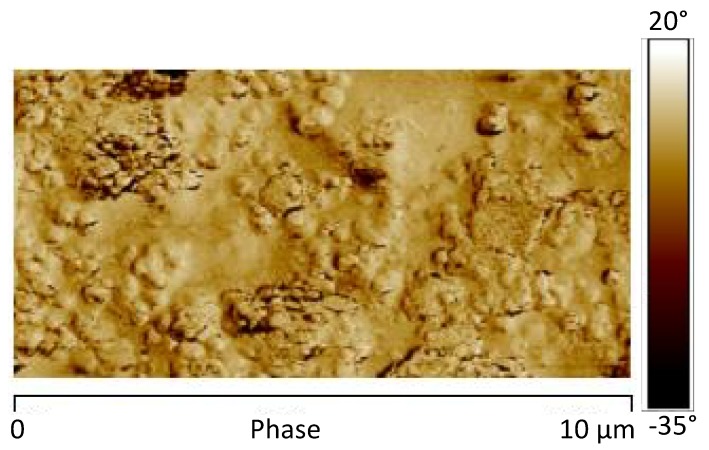
AFM phase map image of the sample 1GNPs (550).

**Figure 19:**
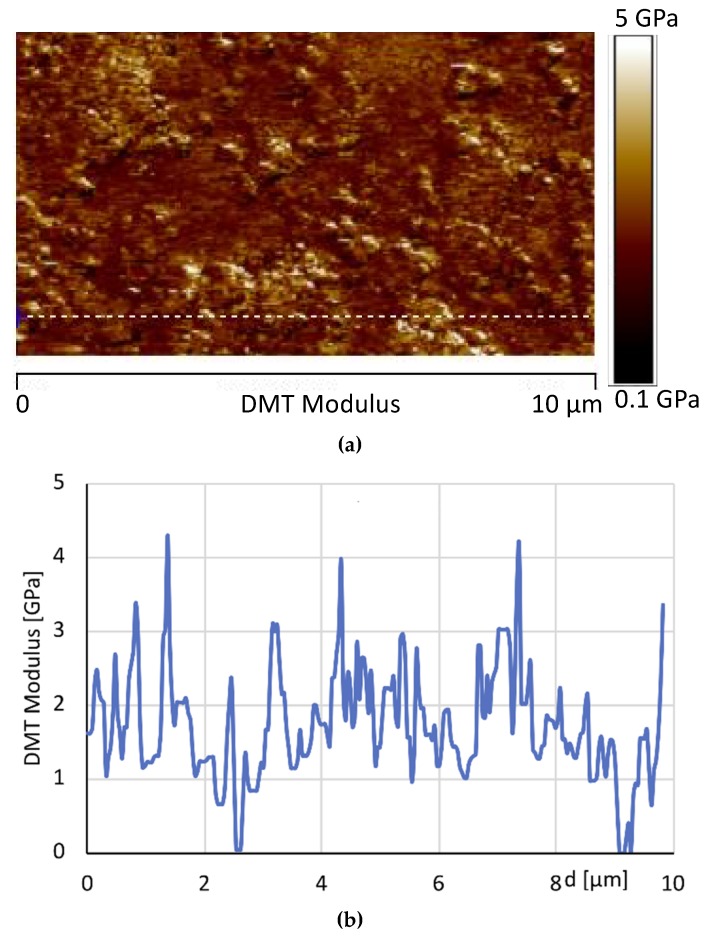
AFM image of the DMT modulus maps (**a**) with the profile of the value of the elastic modulus (**b**) of the same sample.

Figure 18 shows the AFM phase map image of the sample 1GNPs (550) and Figure 19 shows the AFM image of the DMT modulus maps with the profile of the value of the elastic modulus (elastic modulus scale of the sample taken along the white line) of the same sample.

Also, for this sample, as expected, the profile of the value of the elastic is more uniform with respect to that detected for the sample 0GNP. In this case, the effect of the UV irradiation does not determine a decrease in the elastic modulus. It seems that, a slight increase is detected in the region characterized by higher values in modulus. This is most likely due to the effect of the UV irradiation on the thin layer of polymer matrix covering the nanoparticles. The UV irradiation consumes part of the polymer on the GNP nanoparticles; it cannot penetrate beneath them, preventing the polymer matrix from further damages.

It is worth noting that, for each analysed sample, at least five acquisitions over randomly selected areas were considered. The sample surfaces, in terms of morphology and mechanical characteristics, were found to give the same qualitative and quantitative information. Different scan sizes were adopted for the AFM maps reported in the manuscript in order to highlight the morphological characteristics over different areas of each sample. In previous works [59,60], it was demonstrated the capability of the HarmoniX Atomic Force Microscopy technique to draw accurate and reliable micromechanical measurements. Moreover, a multiscale mechanical characterization was performed on a polymeric material, by considering several techniques: Dynamic Mechanical Analysis (DMA, Tritec 2000 DMA, Triton Technology, Mansfield, MA, USA), micro-indentation and HarmoniX Atomic Force Microscopy (AFM) tests, from millimeter to nanometer scale. The AFM determination of surface elastic moduli was successfully compared with that obtained by other techniques, finding good agreement among the results. In a future work, the authors will investigate the complementarity among mechanical characterization techniques, which will be performed at different scales for nanocharged epoxy films. However, a preliminary comparison of elastic modulus obtained by AFM tests and storage modulus obtained by DMA tests at 25 °C have been performed for the initial samples OGNPs and 1GNPs. The AFM elastic moduli have been obtained by averaging the DMT modulus maps, whereas DMA analyses were conducted in tensile mode. The results are shown in Table 2.

The values show a reasonable agreement between techniques operating at different lengths scales.

## 4. Conclusions

In this paper, commercial epoxy-based films, suitable to be used as coatings to protect surfaces of different materials (metals, wood etc.) have been prepared. Graphene-based nanoparticles have been incorporated, at different weight percentages, in the epoxy films. Films (30 ± 1.5 µm thick) unloaded and loaded with graphene-based nanoparticles have been subjected to the accelerated photo-oxidative degradation by exposing them to UV-A radiation, reproducing the ultraviolet (295–380 nm) component of solar radiation at the earth surface. Films without GNPs have proven to be very sensitive to UV treatment. SEM investigation evidences strong damages in the morphological feature of the film surface after the UV treatment. The damages are also reflected in the mechanical performance of the samples. AFM image of the DMT modulus maps of the sample clearly evidences that the values of elastic modulus corresponding to the regions of the epoxy matrix are lower than those detected for the matrix of the sample before UV treatment.

The effects of GNPs on the morphological and structural organization of the samples subjected to the degradation have also been analysed. Graphene Nanoplatelets (GNPs), dispersed in the epoxy-based films, determine a strong decrease in the entity of the damages suffered in the morphological feature of the film surfaces. A simultaneously decrease is observed also in the loss of mechanical performance. Nanometric-resolved maps of the mechanical properties of the film, obtained by AFM investigation, highlight that the dispersion of low percentages, between 0.1 and 1.0% by weight, of graphene nanoplatelets (GNPs) in the films determines a relevant enhancement in the mechanical stability of the irradiated films.

## Figures and Tables

**Table 1:** Sample identification.

Epoxy Matrix (wt.%)	GNPs (wt.%)	UV Irradiation Time (h)	Acronym
0	100	0	GNPs
100	0	0	0GNPs
99.9	0.1	0	01GNPs
99.5	0.5	0	05GNPs
99.0	1.0	0	1GNPs
100	0	550	0GNPs (550)
99.9	0.1	550	01GNPs (550)
99.5	0.5	550	05GNPs (550)
99.0	1.0	550	1GNPs (550)

**Table 2:** Comparison between elastic modulus obtained by AFM tests and storage modulus obtained by DMA tests at 25 °C.

Sample	Modulus (GPa) (AFM)	Modulus (GPa) (DMA)
0GNP	1.1	1.7
1GNPs	2.4	2.6
